# Advancing Applications of System Dynamics in Critical Food Systems Research: Comment on "Using System Dynamics to Understand Transnational Corporate Power in Diet-Related Non-communicable Disease Prevention Policy-Making: A Case Study of South Africa"

**DOI:** 10.34172/ijhpm.9128

**Published:** 2025-07-12

**Authors:** Andrew D. Brown, Simone Sherriff, Benjamin Wood, Steven Allender, Fiona Mitchell, Yin Paradies, Jennifer Browne

**Affiliations:** ^1^School of Health and Social Development, Institute for Health Transformation, Deakin University, Geelong, VIC, Australia.; ^2^Global Centre for Preventive Health and Nutrition, Institute for Health Transformation, Deakin University, Geelong, VIC, Australia.; ^3^Poche Centre for Indigenous Health, University of Sydney, Sydney, NSW, Australia.; ^4^Alfred Deakin Institute, Faculty of Arts and Education, Deakin University, Geelong, VIC, Australia.

**Keywords:** Systems Thinking, System Dynamics, Commercial Determinants, Food Systems, Indigenous Knowledge Systems, Australia

## Abstract

This commentary commends Milsom et al for their critical and rigorous application of qualitative system dynamics to unpack corporate power in food policy-making. Their use of Critical Realism, best practice qualitative methods, and feedback loops exemplifies the maturation of system dynamics applications in public health research. We reflect on how their work aligns with broader debates about power and social theory in system dynamics and how it offers a blueprint for trustworthiness and reflexivity in qualitative modelling. Drawing on our team’s work with Aboriginal communities in Australia, we highlight the value of culturally grounded, participatory modelling in amplifying the voices of communities experiencing historical and ongoing oppression. We support the authors’ call to advance toward simulation modelling and stress the importance of engaging with both system dynamics and community-based knowledge to realise the transformative potential of systems-informed, community-led research in reshaping food policy and practice.

 While applications of systems thinking to public health have existed for a long time,^1^ the UK Foresight Obesity model^2^ marked a turning point where interest in systems approaches to obesity and food systems, especially using system dynamics,^3^ began to grow considerably. The Foresight Model drew together expertise from diverse fields including psychology, physiology, epidemiology, and placed a major emphasis on the production and consumption of food.^2^ Foresight reflected 108 different drivers of obesity and their intersection and reoriented prevention activity to think about obesity in relation to multiple interconnected causes.^2^ A key challenge raised by this early conception was how this complexity could be addressed in practice. Nearly two decades on from the initial Foresight model, the field of food systems research has deepened its application of system dynamics by embracing the core concept of feedback and explicitly considering power in the analysis of food systems.^4,5^ In particular, the increasing use of system dynamics to analyse the commercial determinants of health, defined as “the systems, practices, and pathways through which commercial actors drive health and equity,”^6^ highlights how understanding and engaging with complexity and power can help inform positive changes in food systems to benefit health and prevent disease.^5^ The work undertaken by Milsom and colleagues^7^ is an exemplar of how far the field has progressed in applying system dynamics to understand the complexity of commercial determinants of health.

 When considering the quality of systems approaches to the commercial determinants of health, it is important to acknowledge the extent to which the philosophical position of the research team shapes systems research. In this respect, we fully support the Critical Realist position taken by Milsom and colleagues, as well as the ways in which they embedded this position into their research. Critical Realism follows a realist ontological position as it acknowledges that the world is real and that truth claims can be made with evidence. However, in order to access this reality, researchers are encouraged to go beyond the surface to expose the material world’s real structures and fundamental sources of social control, power imbalances, and inequality.^8^ In their causal loop diagrams, Milsom and colleagues skilfully adopted a power lens to outline the relational and interconnected nature of key actors, practices, institutions, and ideas, which, in totality, impede transformative policy action on non-communicable diseases in South Africa. In doing so, the authors went “beyond the surface” to expose key upstream and structural drivers of poor health and health inequities (eg, institutional arrangements that reinforce the economic and political power of ultra-processed food corporations), and to identify potential leverage points for systems transformation (eg, eliminating agricultural subsidies that promote the unsustainable production of cheap inputs for ultra-processed foods). Yet Critical Realism also adopts a subjectivist epistemological position by acknowledging that reality is difficult to access directly, and that a researcher’s inquiry into reality can easily become distorted.^9,10^ We commend the approach taken by Milsom and colleagues in dealing with the subjective nature of portraying the material reality of the system in question, including their use of best practice qualitative methods, and their detailed reflection on how their background and beliefs may have influenced their research.

 While the work by Milsom and colleagues represents an exemplar of explicitly applying a critical perspective to system dynamics in food systems research, the field of system dynamics has long grappled with the concept of power and its relationship to systems change. Extensive discussion has been carried out in the system dynamics literature about the voluntarism/determinism debate and whether system dynamics aligns with social theories of “regulation” that emphasise the cohesiveness of society and maintaining the status quo or “radical change” theories that emphasise societal conflict in which power is used to dominate.^3^ Lane ultimately concludes that system dynamics can be applied across this spectrum of social theories but emphasises the need to be considered and explicit in which theories are being applied and why in modelling efforts. It has been recognised for over a decade that the expertise of the field of system dynamics is underutilised in public health applications^11^ and, more recently, for analysing commercial determinants of health.^5^ Considering how Milsom and colleagues make their critical position clear, strengthening their application of system dynamics, broadens this call to encourage broader engagement with the field, not just on best practices for modelling or participation in modelling, but also in considering its theoretical foundations and contributions.

 System dynamics has a rich history of participatory modelling, and explicit calls of considering power in this space.^1^ In documenting their methods for modelling of the diet-related non-communicable disease-prevention policy-making system, Milsom and colleagues reflect many best practice principles in qualitative research, enabling consideration of power and participation. Various criteria, such as credibility, transferability, dependability, confirmability and reflexivity have been recommended to improve the quality and transparency of qualitative research.^12^ Although the applicability of these quality criteria has been debated by “Big Q” qualitative scholars,^13^ strategies to ensure trustworthiness could be strengthened in qualitative system dynamics studies in public health, demonstrated by calls for more focus on modelling processes rather than just the resulting model.^11^ This article, however, provides a detailed description of the context, sampling strategy, data collection and analysis process, and the steps used to develop combined causal loop diagrams. The credibility of the resultant system models was further enhanced through documented ongoing participant engagement via interviews, model conceptualisation and model validation. Importantly, the lead author provided a comprehensive reflection on how the researchers’ positionality may have influenced the research question, data collection analysis and interpretation of findings. It is unfortunate that these reflexive considerations were relegated to the Supplementary material, presumably due to word count limitations. Taken together this article provides a valuable blueprint for undertaking rigorous qualitative system dynamics research within food systems research, with opportunities to further engage with the broader system dynamics literature.

 For over a decade, our team has developed our own capacity and relationships with communities to apply system dynamics to food systems.^4^ Our experience includes applying qualitative system dynamics methods in Aboriginal food and nutrition policy research in Australia, particularly in food systems work that directly engages with power and the commercial determinants of health.^14^ We have found that, if applied in a culturally safe manner that supports Aboriginal leadership, systems thinking approaches have potential synergies with Aboriginal worldviews and opportunities for bringing Aboriginal and Western knowledge systems into conversation.

 Using group model building (GMB), the key method that system dynamics uses to build models in partnership with communities, can enable key principles of co-creation to be upheld, such as sharing of power; privileging and valuing local Aboriginal knowledges; and empowering the community to define the system and co-create policy actions.^15^ For example, our Food Policies for Aboriginal and Torres Strait Islander Health study demonstrated that GMB was useful for elevating Aboriginal voices in food policy dialogues and developing a shared understanding of the system-level drivers of food choice.^14^ Like Milsom and colleagues, we also found this approach helpful for uncovering the concentration of industry power and influence in the system, which we will continue to interrogate in our new program of research on the commercial determinants of Indigenous health.

 Applying GMB in practice in a way that built trust and led to deep insights required a long-term process. Our work has spanned over the course of several years and began with exploring tools and practices from GMB with a small group of Aboriginal researchers and health practitioners with whom we have existing relationships and trust. Key to this initial work was avoiding assumptions that GMB or systems thinking more generally were already a meaningful set of methods or tools to work with Aboriginal communities but rather being open to the answer that they may not be. Additionally, applying “two-way learning” was key to ensure Aboriginal Ways of Knowing, Being, and Doing actively shaped this process. This focused, intentional work grew to engage a larger number of Aboriginal scholars, practitioners, community members, and Elders. A key principle in the growth of this work was that it was led by Aboriginal people who were comfortable facilitating GMB sessions, with support from a team, with a larger segment of community members. As we continued to explore ways of putting systems thinking into practice in partnership with Aboriginal communities, it was important that we took a long-term, relationship-based view, where we aimed to work together across multiple projects, rather than discrete pieces of work. All of this took a lot of time, trust, and willingness to acknowledge mistakes along the way. Further work is required to understand how to intertwine systems thinking more closely with Indigenous knowledge systems such as pattern thinking,^16^ such as in this recent food systems research by Glassey et al in the New Zealand context.^17^

 One of the key next steps identified by Milsom and colleagues is to move from qualitative modelling to quantitative simulation modelling. Simulation modelling is a core method in system dynamics, and while simulation has already been applied to public health generally and food systems in particular, the growing interest in extending the use of simulation is an exciting opportunity and in line with the broader field of system dynamics. Like Milsom and colleagues, we plan to use system dynamics to analyse interview data and academic literature, ultimately extending our qualitative mapping to quantitative simulation modelling to evaluate the system-level impacts of Aboriginal-led policy-priorities. We echo the calls to deeply engage with the system dynamics literature and field as applications of system dynamics continue to spread^1,11^ and advance in commercial determinants of health research.^5^ Considerations of power and critical reflexivity must extend into quantitative modelling work. The limitations of what can be meaningfully represented quantitatively, and the process of translation from qualitative methods to quantitative methods must be recognised as a subjective process, with a critical lens applied consistently throughout. Considerations of what data are used or not, who owns the data, and what data are available are key considerations in the quality of a resulting model, the meaningfulness of its results, and whether it contributes to advancing structural change necessary to address colonisation and the commercial determinants of health.

 The work of Milsom and colleagues exemplifies the opportunity not just to engage with literature about how to build and document models, but to also consider system dynamics insights about process, social theory, and power. Beyond engagement with system dynamics literature, it is crucial to engage with literature, and knowledge more broadly, that is important to the communities we partner with in understanding and changing food systems. Using both qualitative and quantitative system dynamics, with explicit consideration of power and community leadership in modelling processes, offers an opportunity to address the commercial determinants of health and transform food systems.

## Ethical issues

 Not applicable.

## Conflicts of interest

 Authors declare that they have no conflicts of interest.
